# VALIDATION OF THE CAREGIVER REACTION SCALE IN A SAMPLE OF NON-HELP-SEEKING CAREGIVERS

**DOI:** 10.1093/geroni/igz038.2506

**Published:** 2019-11-08

**Authors:** Kelly A O Malley, Kelsey Bacharz, Sara H Qualls

**Affiliations:** 1 New England GRECC, Boston VA Healthcare System, Boston, Massachusetts, United States; 2 University of Colorado Colorado Springs, Colorado Springs, Colorado, United States

## Abstract

The Caregiver Reaction Scale (CRS) is a comprehensive measure of the family caregiving experience that assesses burden, family strains and positive aspects of caregiving (PAC). The CRS has been validated in sample of older adult help-seeking caregivers, but its validity and reliability in a non-help-seeking sample of caregivers was unknown. This study aimed to explore how well the CRS assesses the full caregiving experience in a younger non-help-seeking sample of family caregivers and to further evaluate the validity of the PAC subscales. A sample of non-help-seeking caregivers (N =452; Mage = 48.56, SD = 17.15) completed online questionnaires of burden, positive aspects of caregiving, and psychological well-being. All subscales of the CRS demonstrated very good internal consistency reliability (α ≤ .88). The PAC subscales of the CRS demonstrated medium to large positive correlations with a measure of positive aspects of caregiving (r ≥ .44) and small to medium positive correlations with psychological well-being (.25 ≥ r ≤ .42). Burden subscales of the CRS had large positive correlations with another measure of burden (r ≥ .66). Medium positive correlations were also found between family and job conflict subscales of the CRS and the burden measure (r ≥ .35). CRS PAC subscales were negatively correlated with the burden measure (r ≤ -.13). The CRS is a valid and reliable measure of the caregiving experience as evidenced by convergent and discriminant validity of CRS subscales and well validated measures of burden and positive aspects of caregiving.

